# Supplementary material for: High-resolution neural recordings improve the accuracy of speech decoding
Source: Nat Commun. 2023 Nov 6;14:6938. doi: 10.1038/s41467-023-42555-1 (PMC10628285; doi:10.1038/s41467-023-42555-1)
Supplement: Supplementary file 1 — Supplementary Information [file 41467_2023_42555_MOESM1_ESM.pdf]

Supplementary Materials for

High-resolution neural recordings improve the accuracy of speech decoding

Duraivel *et al.*

*Corresponding authors. Email: gregory.cogan@duke.edu , j.viventi@duke.edu

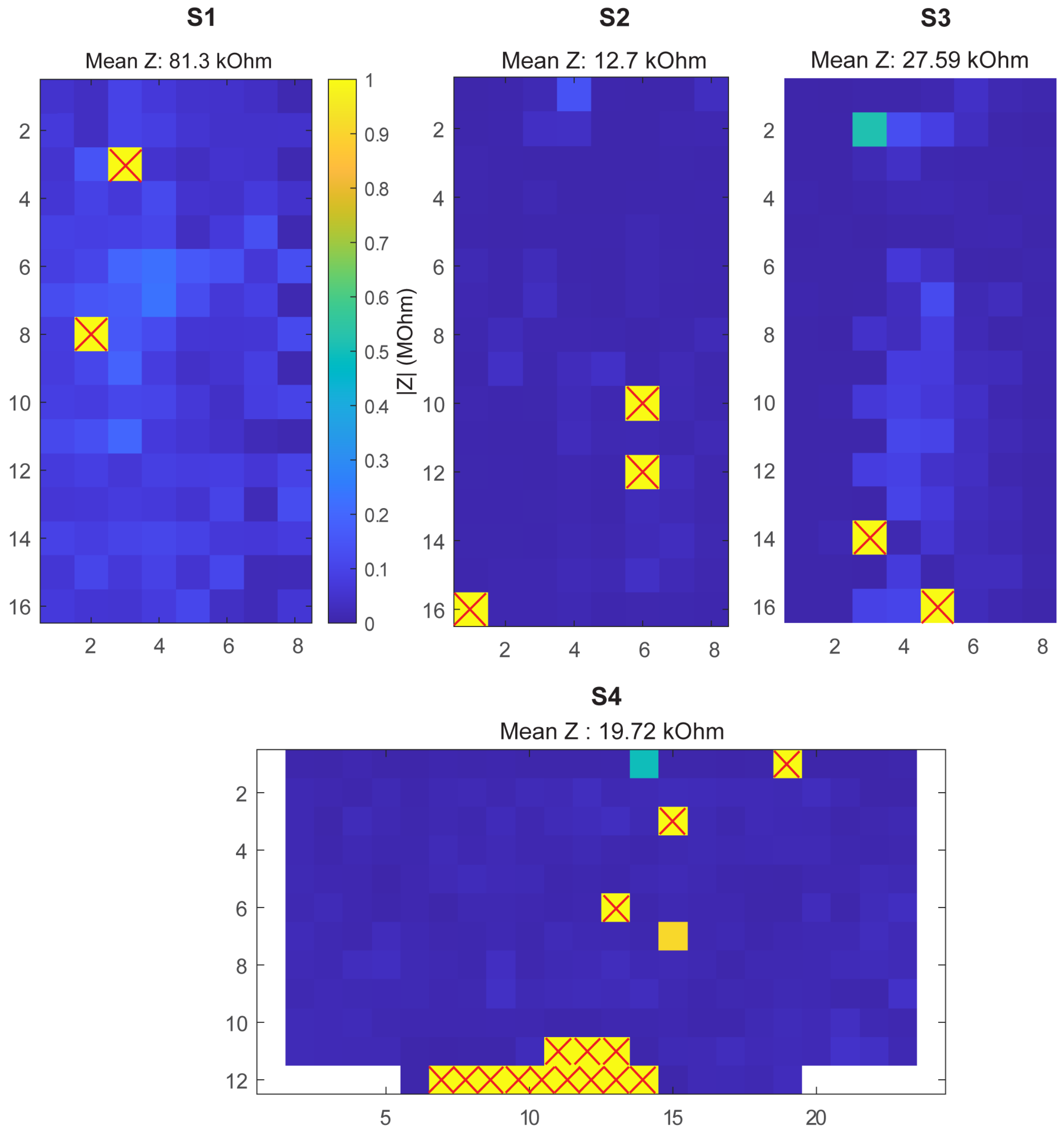

Supplementary Figure 1. Spatial maps of *in vivo* electrode impedance during intraoperative μECoG implantation. Uniform impedance measurement at 1kHz for 128-channel (S1, S2, and S3) and 256-channel (S4) LCP-TF electrode arrays demonstrate our successful implantation procedure in the intraoperative setting. Channels with impedance >1 MΩ were considered non-functional and are identified with red markers in the spatial maps.

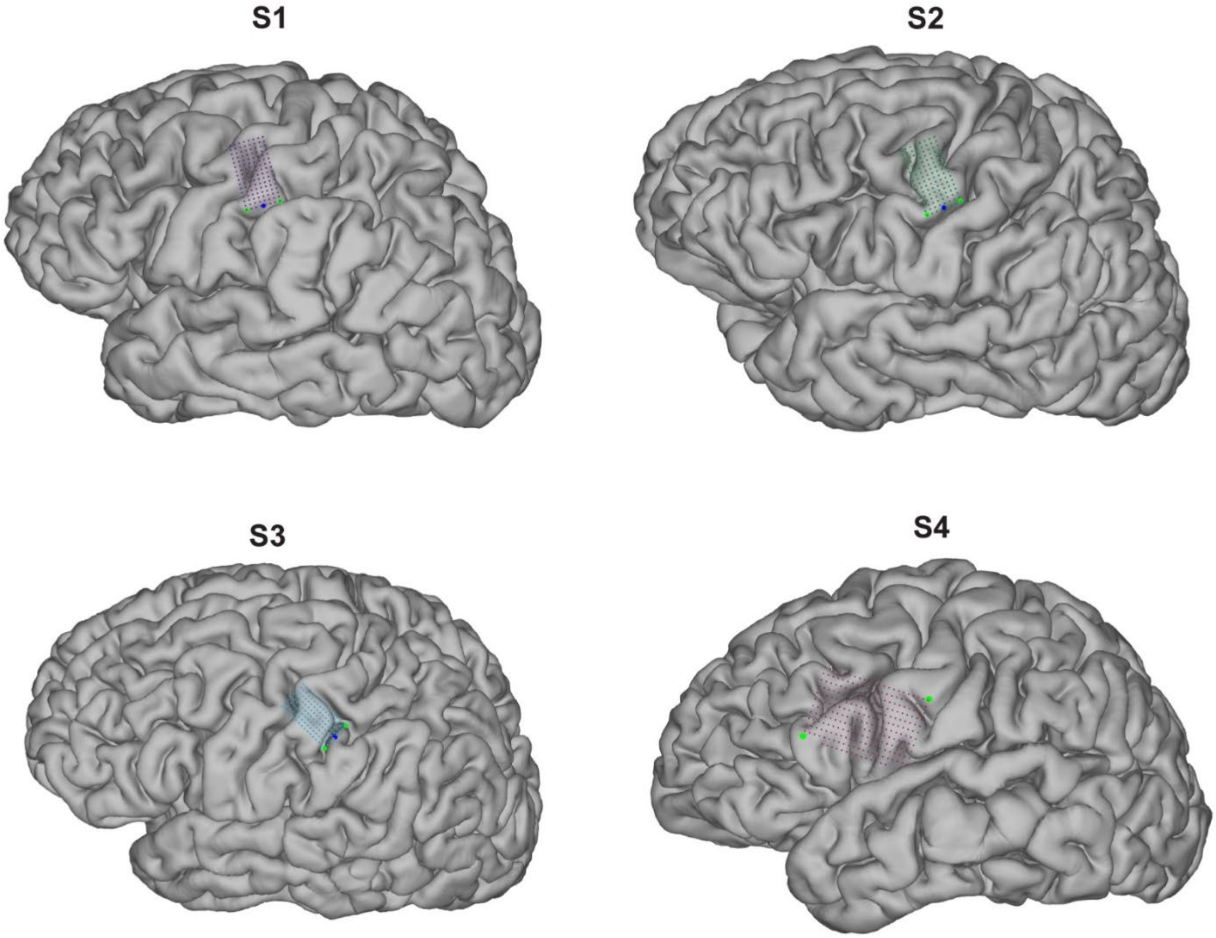

Supplementary Figure 2. Sensorimotor target locations of intraoperative µECoG recordings during awake speech task. For 128-channel electrode arrays (S1, S2, and S3), green and blue pointers indicate array markers from intra-operatively acquired CT scans post implantation. 256-channel (S4) electrode array was localized using intra-operatively acquired BrainLab markers, along the diagonally opposite corners.

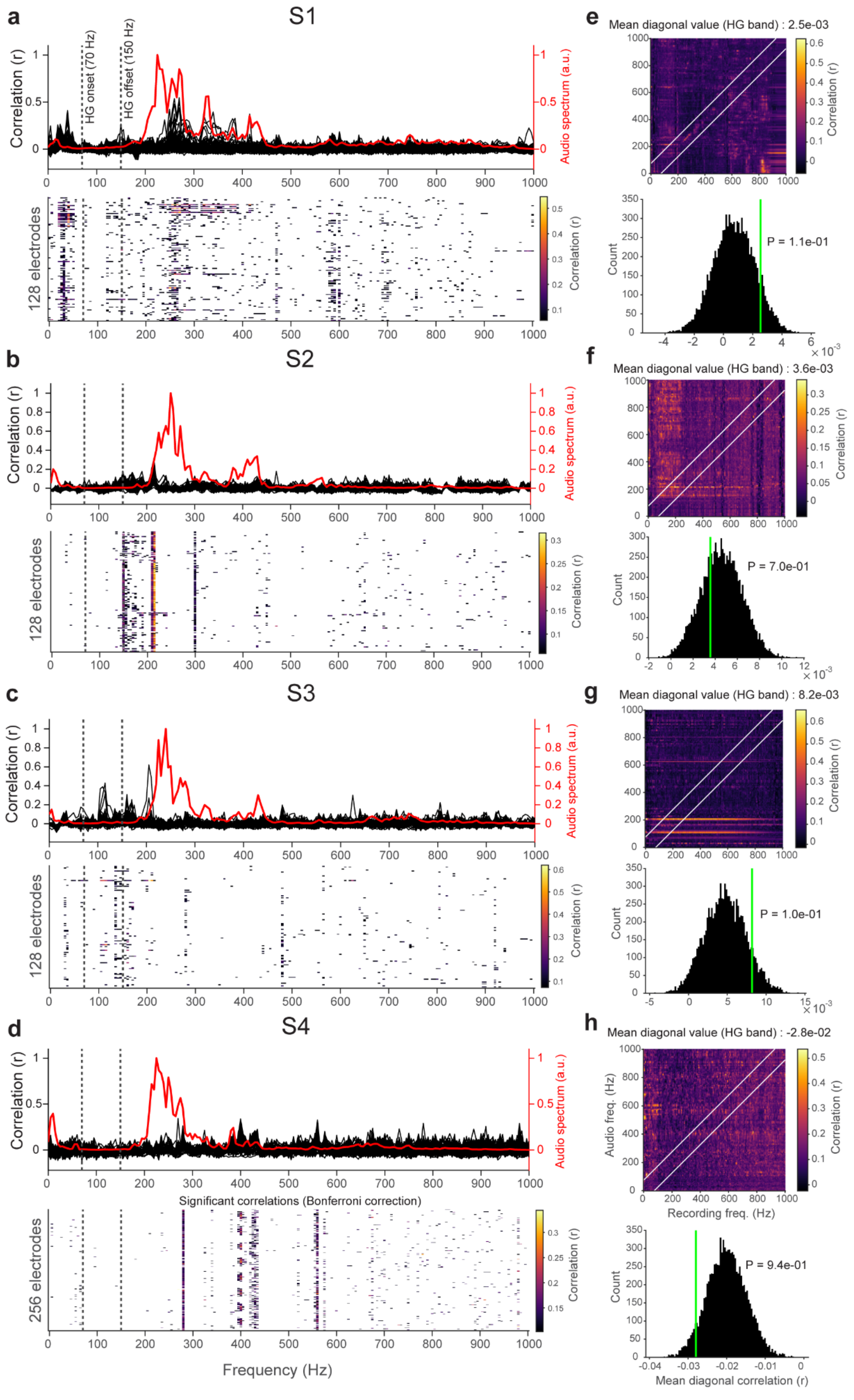

Supplementary Figure 3. Detecting acoustic contamination of neural signals: (a-d) Correlation between the audio stimulus and µECoG signals in subjects S1-S4. The red curve represents the mean PSD of the audio signal, and each black trace indicates the correlation coefficients between the spectrograms of the electrodes and audio signal. Heat maps indicate correlation values across all electrodes and frequency bins. **(e-h)** Objective quantification of contamination for 4 subjects. Each heatmap indicates the mean audio-neural contamination matrices across electrodes. The mean of diagonal values in the high-gamma band (HG: 70-150 Hz) is compared to the distribution of 10,000 shuffled diagonal values (green bars indicate non-significant contamination). Electrode-specific analysis indicate absence of contamination in the HG band across all electrodes in all subjects.

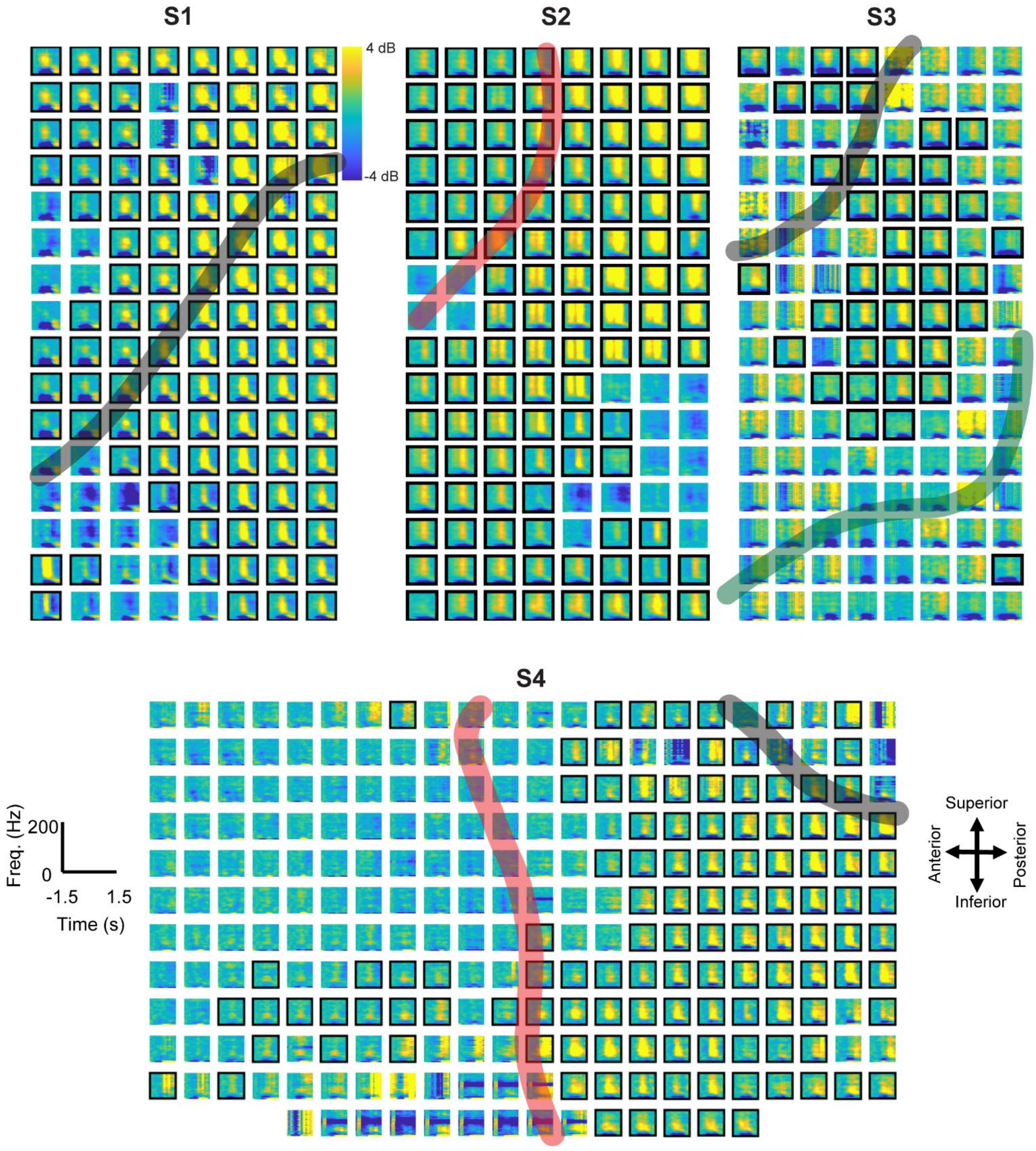

Supplementary Figure 4. **Spectrogram activations for four intraoperative subjects during a speech production task**. Normalized spectrograms reveal distinct spectro-temporal modulation for each μECoG channel. We focus on the significant rise in HG power during speech production (black border: one-sided permutation test, $p < 0.05$; corrected via false discovery rate method). Shaded red, grey, and green curves indicate the locations of pre-central, central, and post-central sulcus, respectively. Electrodes with significant HG modulation reveal the spatial activation profile across the μECoG array for all subjects (S1: 111/128, S2: 111/128, S3: 63/128, and S4: 149/256).

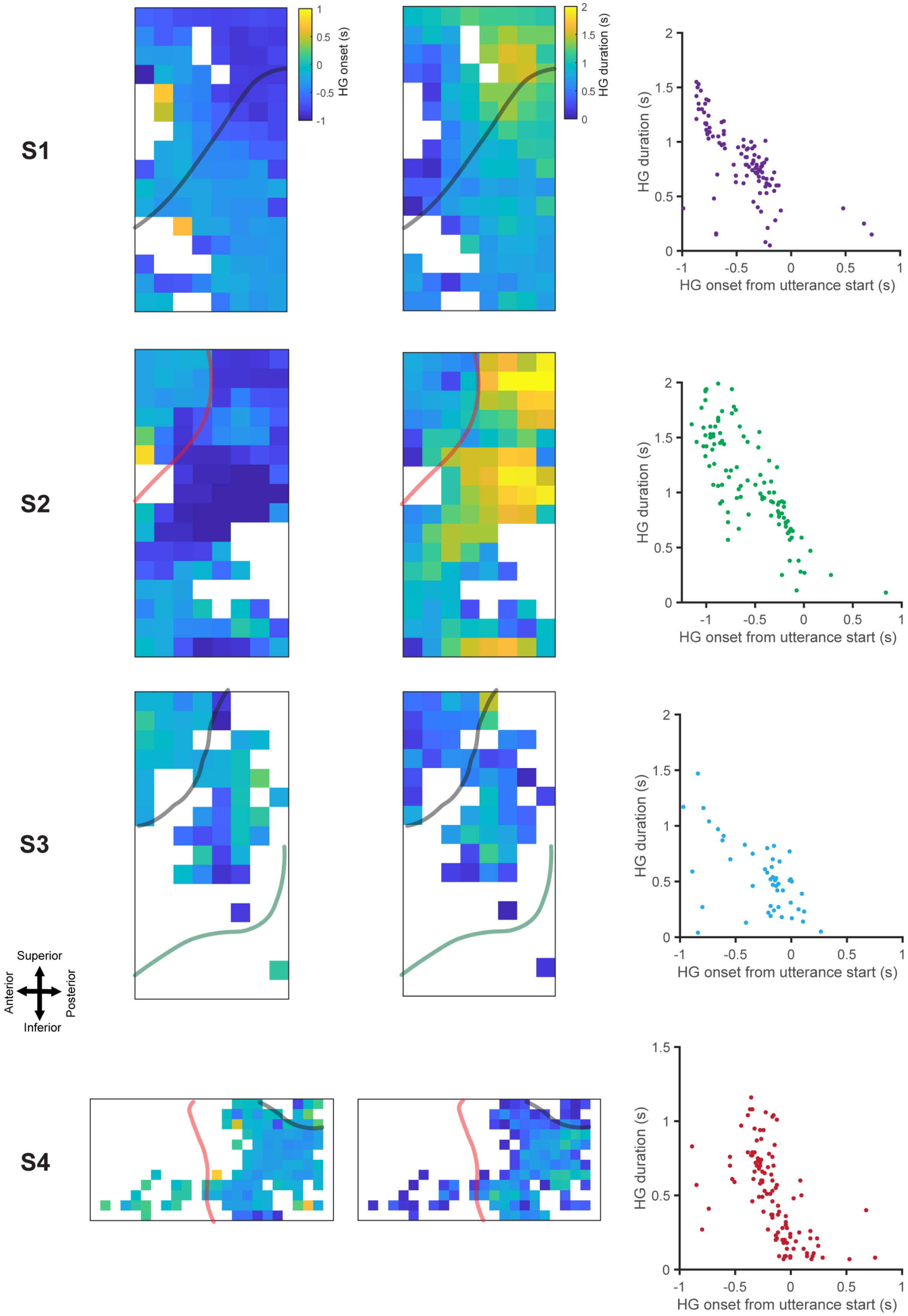

Supplementary Figure 5. Morphological characteristics of speech-evoked high-gamma activations. Spatial layout of HG onsets and duration reveal fine-scale spatio-temporal activation of speech neural activations in each µECoG channel. HG onsets (**left column**) and duration (**middle column**) were calculated using cluster-correction based one-sided permutation test ($p < 0.05$, 10000 iterations). Non-significant channels were masked with white space. Shaded red, grey, and green curves indicate the locations of pre-central, central, and post-central sulcus, respectively. **Right column:** Electrodes with earlier onsets had extended duration, indicating neural evidence for speech preparatory characteristics.

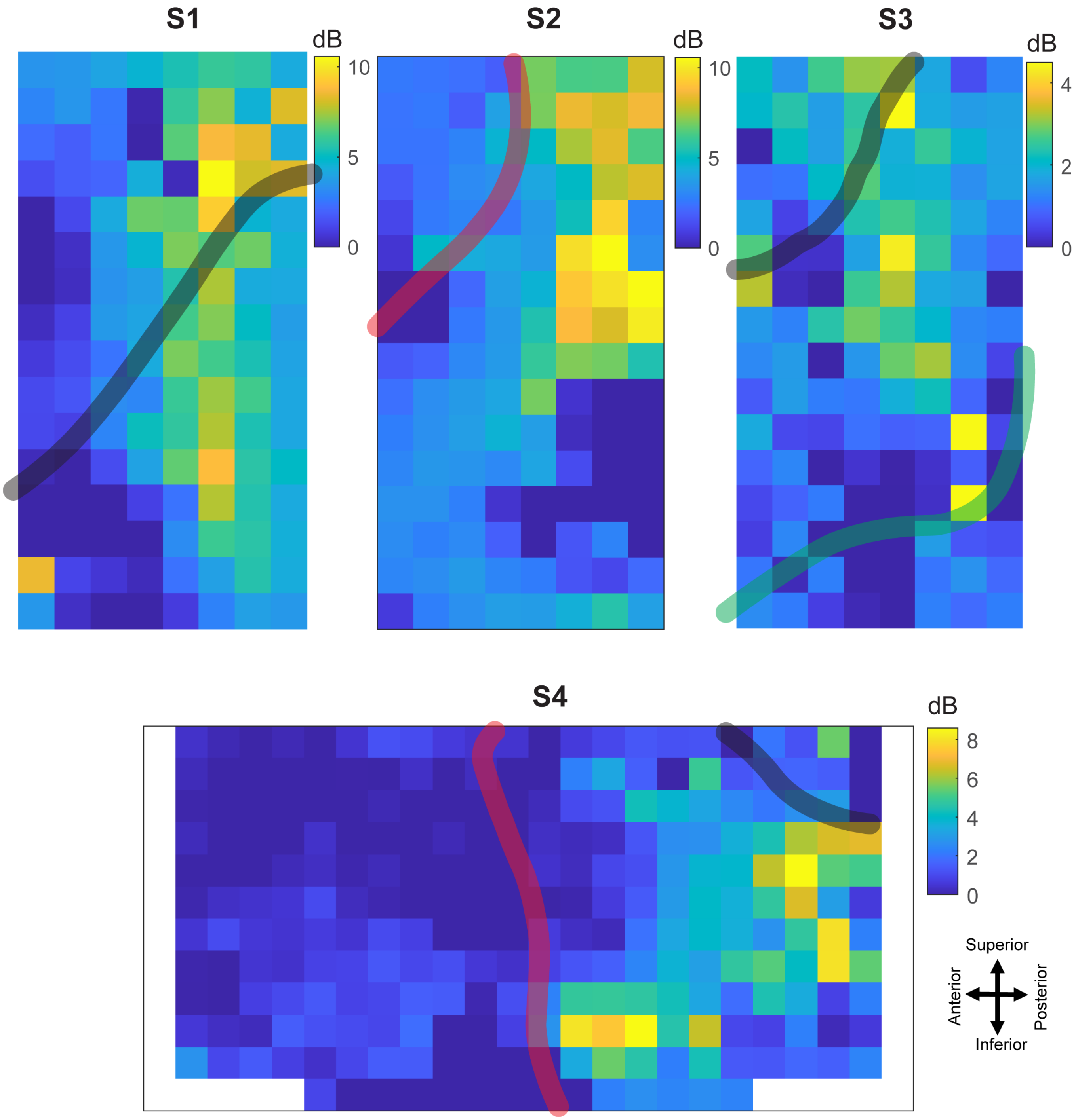

Supplementary Figure 6. **High-Gamma ESNR for four intraoperative subjects during speech**. Spatial layout of HG power in decibels (dB) reveal fine-scale tuning of speech neural activations in each µECoG channel (median HG ESNR = 2.9 dB, S1: 4.2 dB (max: 10.1 dB), S2: 3.5 dB (max: 10.1 dB), S3: 1.9 dB (max: 4.2 dB), and S4: 2.1 dB (max: 8.2 dB)). Shaded red, grey, and green curves indicate the locations of pre-central, central, and post-central sulcus, respectively.

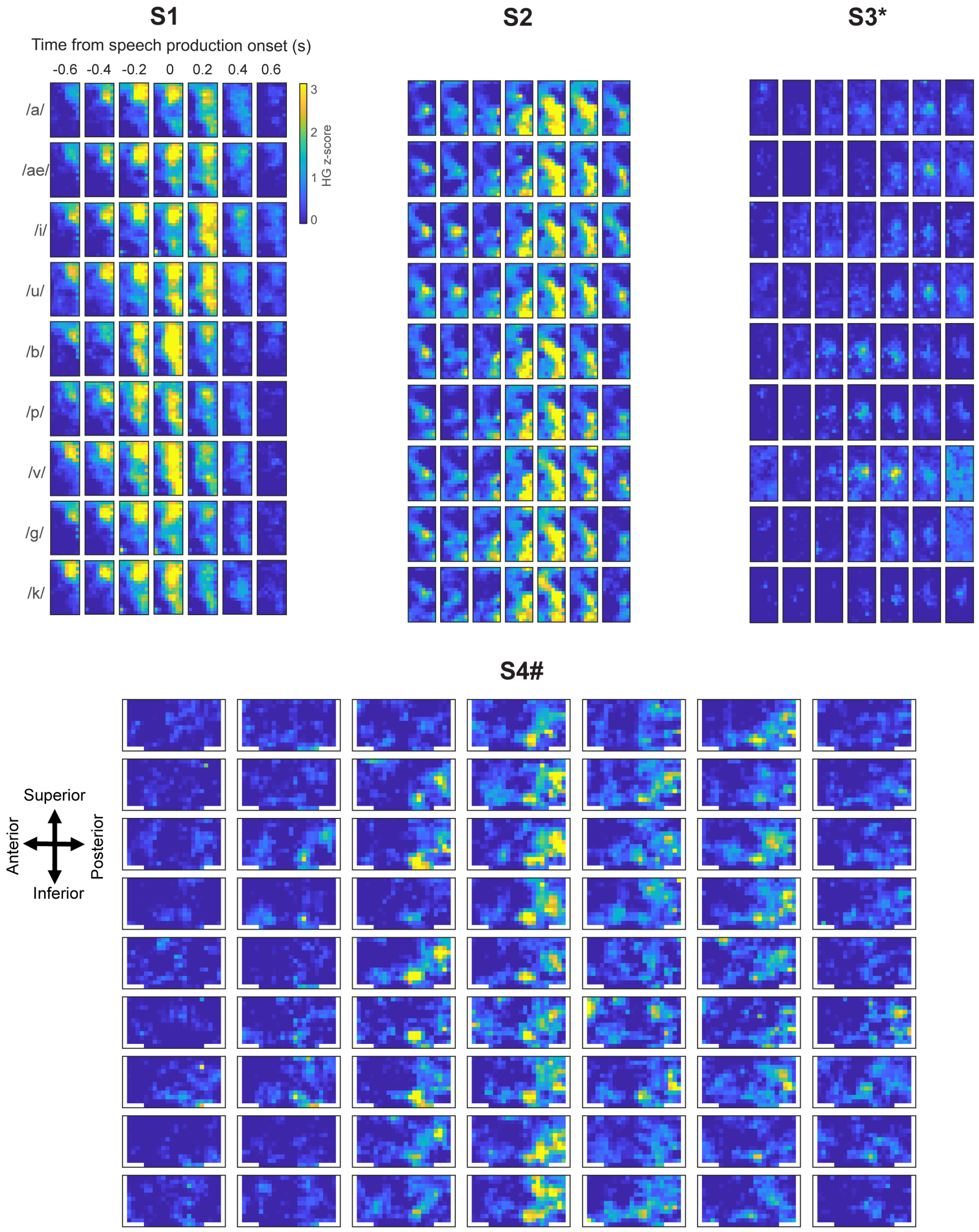

Supplementary Figure 7. **Fine-scale spatiotemporal activations of HG power during speech production tasks**. Channel map activations reveal distinct, highly resolved spatio-temporal activity for all subjects during each phoneme utterance (first-position phonemes). Phonemes with similar articulatory features exhibit similar activation patterns at speech utterance. Subtle differences in spatial patterns of activation and relative HG power can be observed between phonemes that differ by one articulatory feature (/b/ vs. /p/, and /g/ vs. /k/). S1 and S2 exhibited stronger HG-SNR revealing clear phoneme-specific activation patterns. S3 and S4 exhibited weaker yet significant activations due to low SNR (*) and recording duration (#), respectively (* - low SNR recording, # - completed 1 block only).

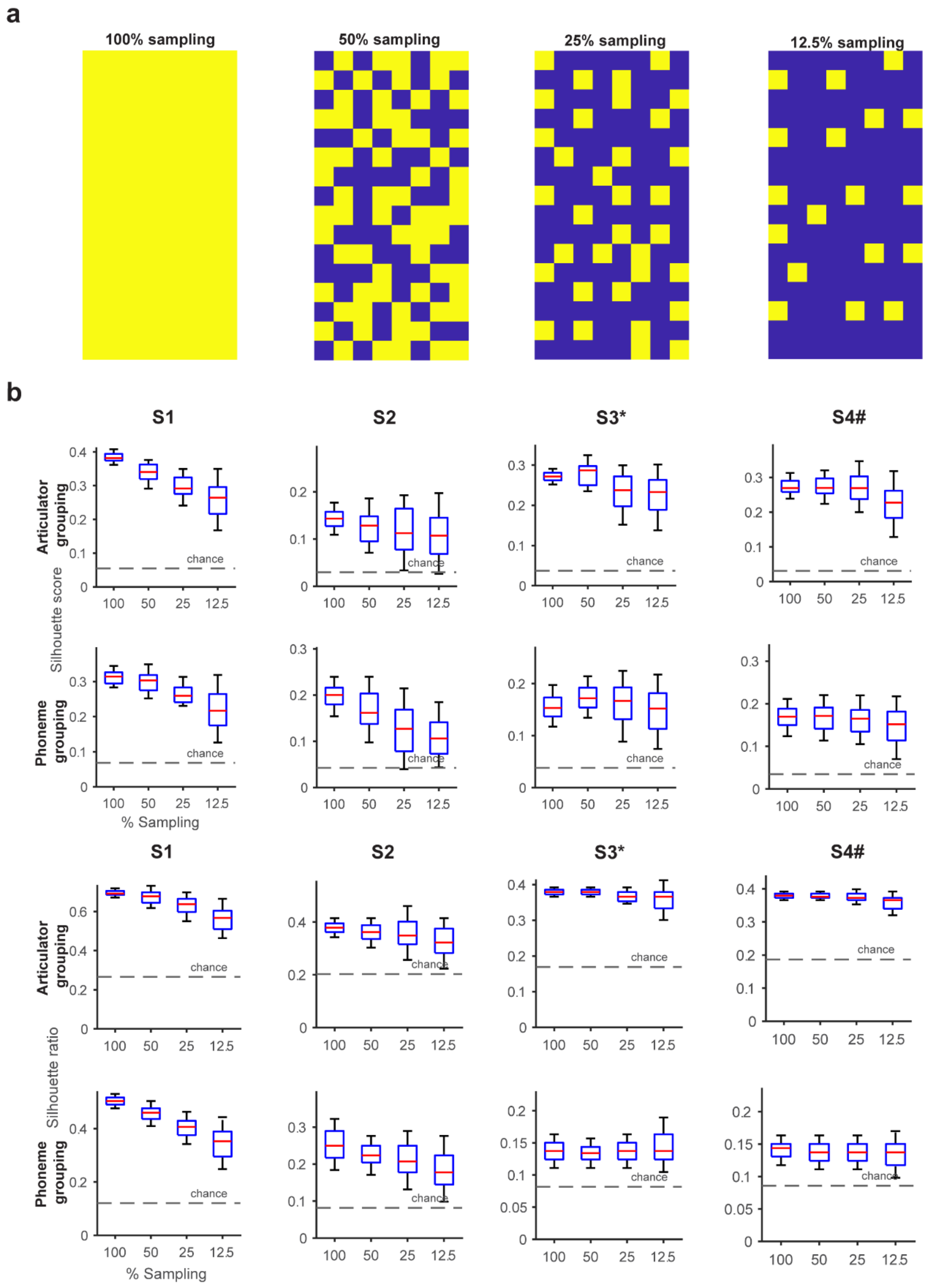

Supplementary Figure 8. **High resolution recording enables robust cortical state-space for phoneme articulators in SMC.** **a** Demonstration of electrode subsampling using Poisson-disc method. Subsampled electrodes are uniformly distributed across the array. This method of uniform subsampling provides valid low-resolution simulation when compared to random sampling of electrodes. **b** Silhouette score and silhouette ratio measures clustering of utterance trials in the cortical state-space (SVD – tSNE) with respect to either articulatory or phoneme grouping (* - low SNR recording, # - completed 1 block only). Spatial subsampling of electrodes using Poisson-disc sampling at fixed spatial coverage deforms the cortical-state-space for all subjects, as measured by a decrease in silhouette values. Each distribution contains state-space evaluations from 50 electrode sub-samplings (n = 50). The red lines and blue boxes indicate the median and 25/75th percentile, and the dotted lines indicate the full range of the distributions. High-resolution cortical sampling enables accurate capturing of speech articulatory features in SMC.

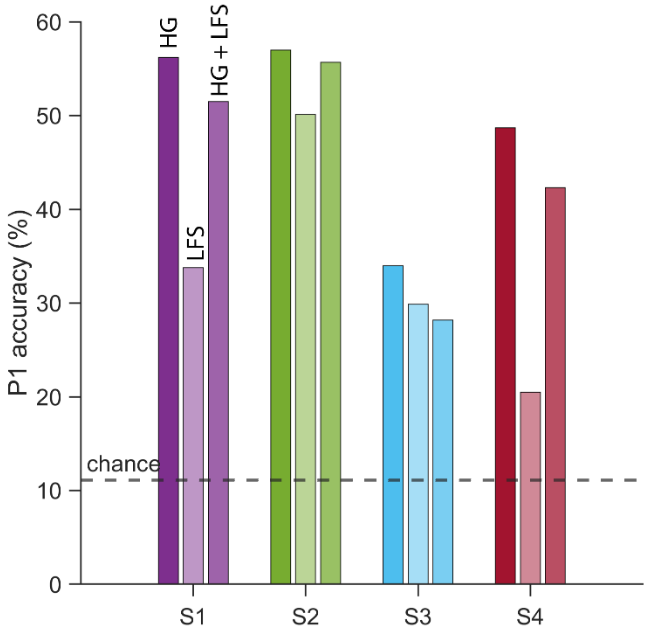

Supplementary Figure 9: Phoneme decoding was accurate for both high-gamma (HG) and low-frequency signals (LFS). HG power achieved higher decoding compared to LFS for all four subjects and combining of HG and LFS resulted in comparable performance to HG alone.

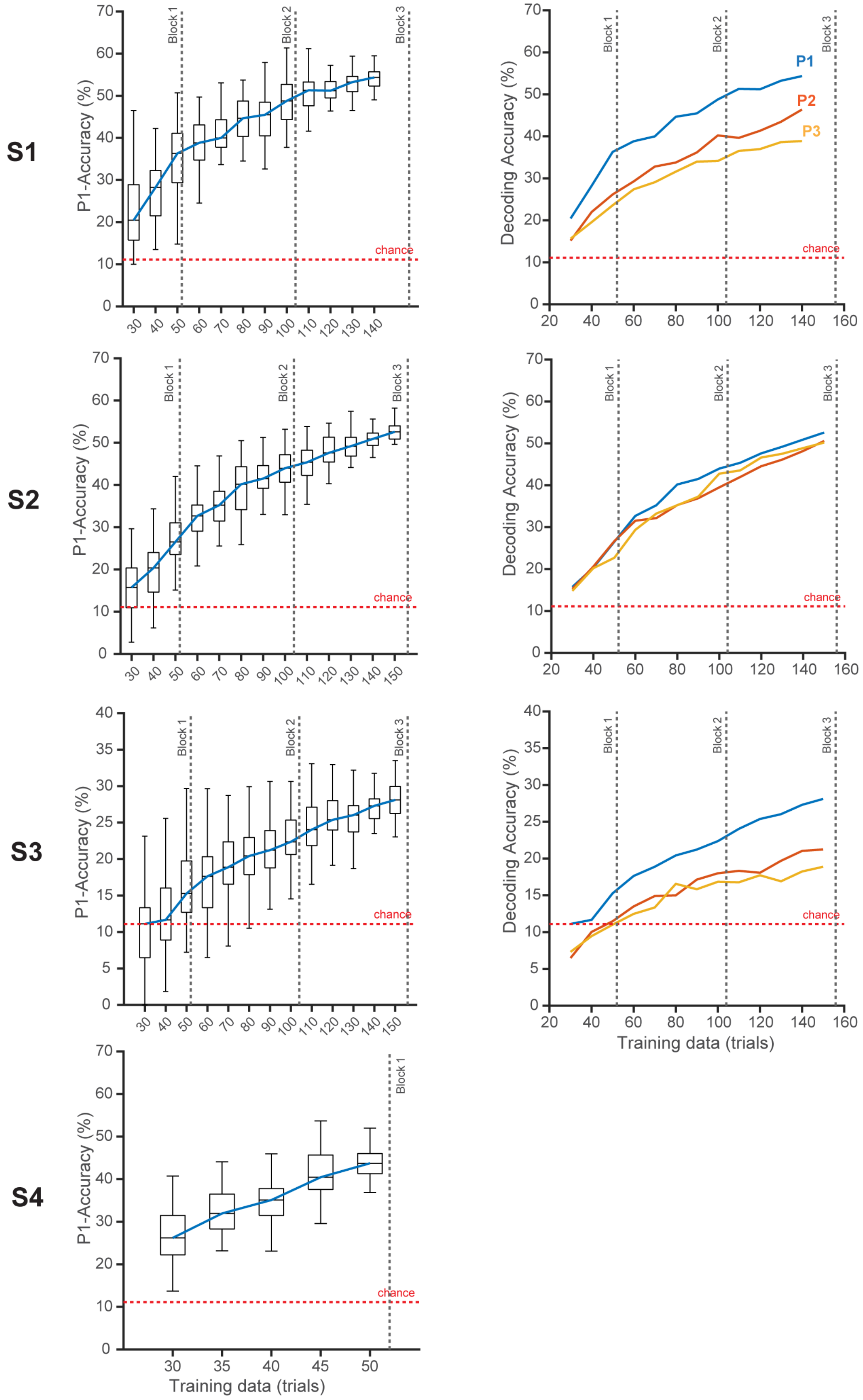

Supplementary Figure 10. **Effect of training duration on decoding performance for all four subjects**. To investigate the impact of training duration, decoding performance is calculated on subsampled trials (n = 50 subsample trials) by maintaining equal distribution of phoneme trials in each sampling. The horizontal center lines and the boxes indicate the median and 25/75th percentile and vertical solid lines represent the full-range of the distribution. The decoding performance (**left column**) increases with increased data for all subjects, as indicated my median decoding accuracy. The vertical dotted lines indicate the number of trials the patients would have completed by the end of an experimental block. These performance trends were stable across all phoneme positions as indicated by median decoding performance for P1, P2, and P3 (**right column**). Subject S4 completed one-block of speech experiment, thereby, limiting the number of trials required to perform subsampling analysis for P2 and P3.

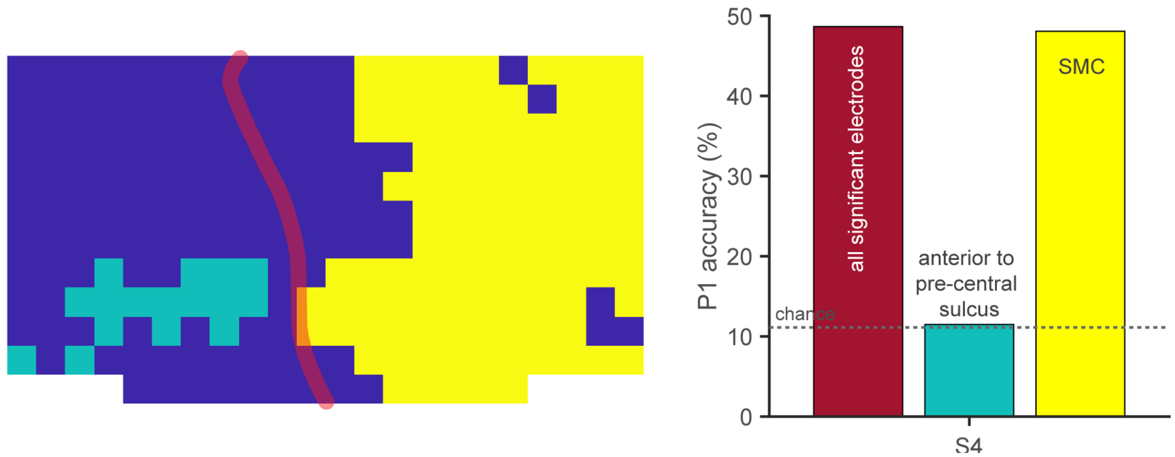

Supplementary Figure 11: Decoding characteristics of S4. 256 channel recordings from S4 enabled HG activations from both SMC (yellow; 108 electrodes) and inferior frontal regions (16 electrodes; cyan). Successful decoding was specific to electrodes over SMC (accuracy; 48%) when compared against electrodes anterior to pre-central sulcus (accuracy: 11.5%). Shaded red curve indicates the anatomical location of the pre-central sulcus.

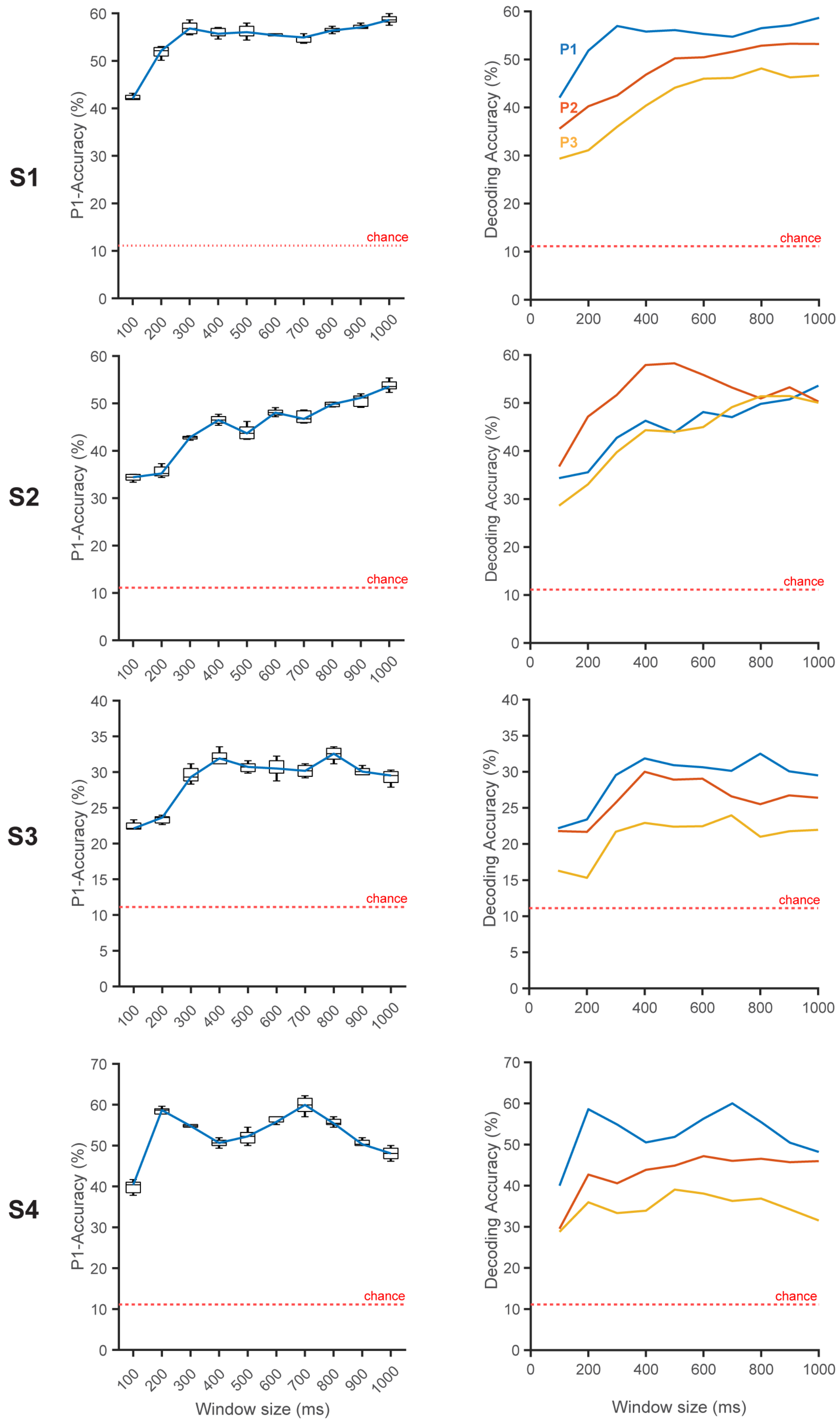

Supplementary Figure 12. **Effect of time-window on decoding performance for all four subjects**. To investigate the amount of time necessary to decode each phoneme, decoding performance is calculated on varying time-window (n = 10 repetitions). The horizontal center lines and the boxes indicate the median and 25/75th percentile and vertical solid lines represent the full-range of the distribution. The decoding performance (**left column**) increases with longer windows until saturation. The saturation point is specific for each subject and each phoneme position (**right column**; median decoding performance for P1, P2, and P3), indicating the evidence for subject-specific neural tracking of phoneme sequences during speech utterance. The reduction in performances after saturation for S3 and S4, were likely due to neural contamination from adjacent phonemes, exacerbated by low SNR and recording duration, respectively.

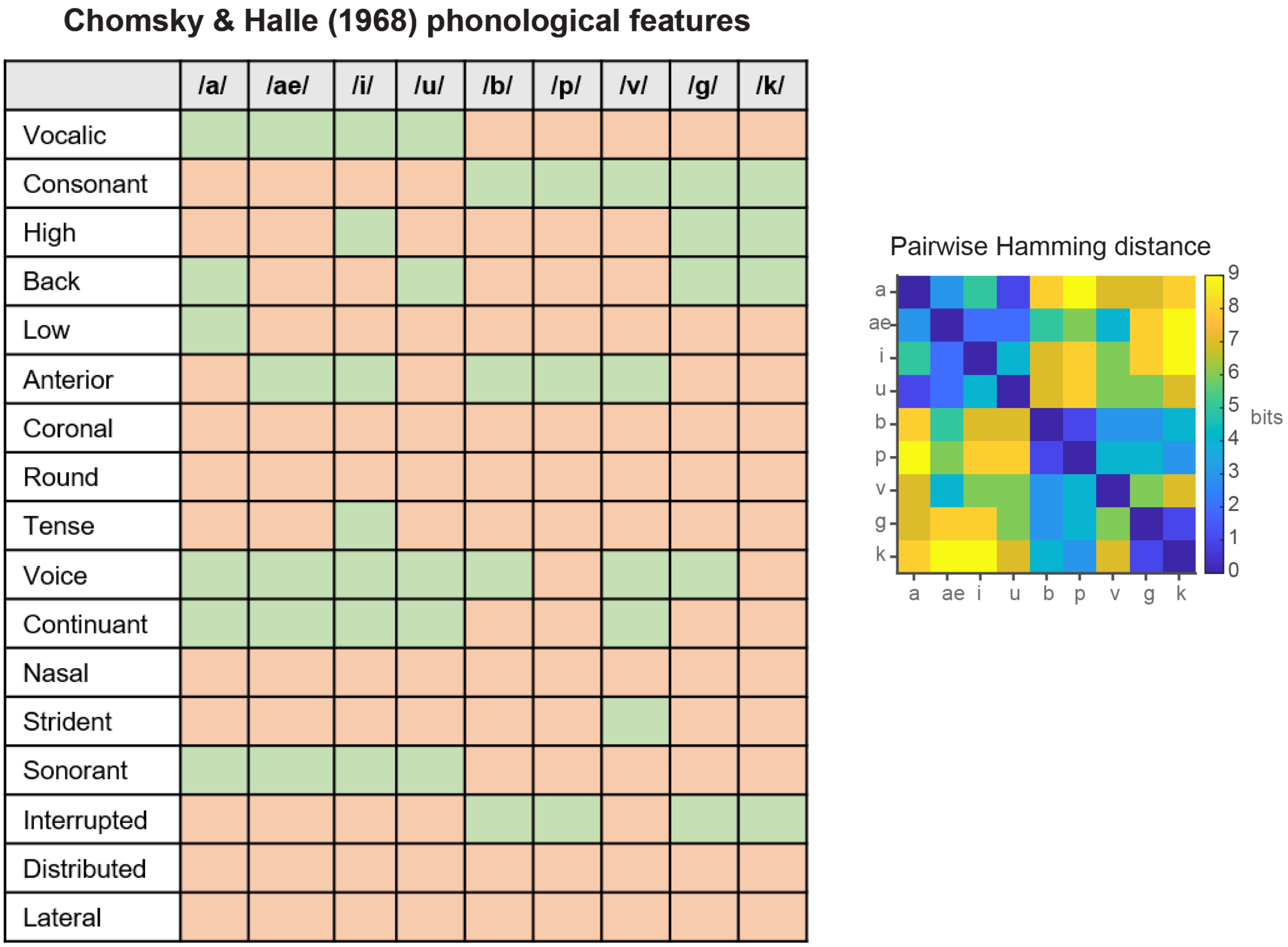

Supplementary Figure 13. Chomsky & Halle (1968) phonological features for phonemes used in our study. Each phoneme is identified by 17-bit binary feature vector (green = 1, red = 0) to characterize phonological articulatory features. A pairwise distance metric was computed between all possible phoneme pairs using Hamming distance. The resultant phonological distance values were lower for phonemes with similar phonological features and vice-versa (e.g., the phoneme /p/ differed from /b/ by 1 bit, and from /g/ by 5 bits).

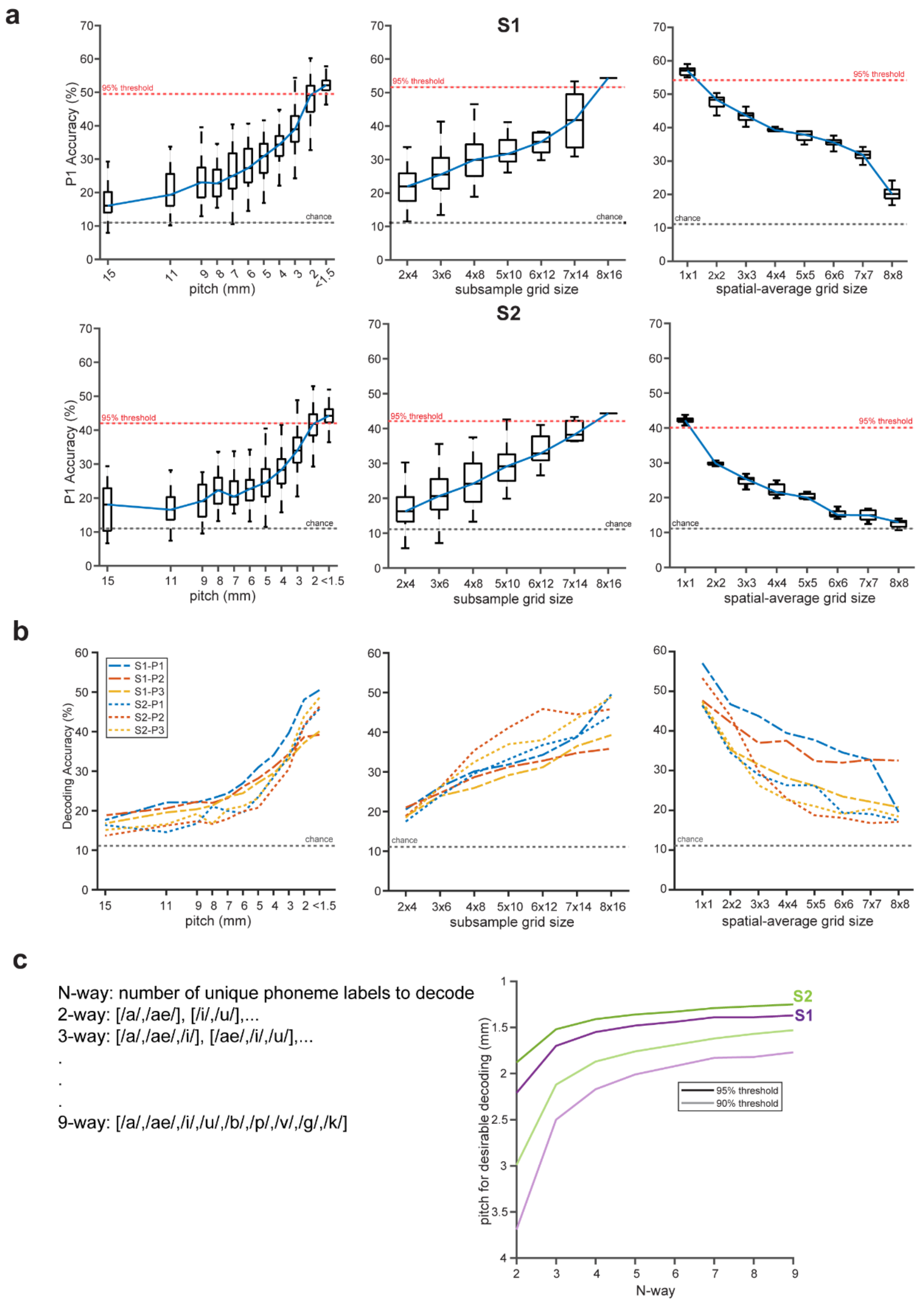

Supplementary Figure 14 Effect of spatial-resolution, coverage, and micro-scale contact size on decoding performance for both S1 and S2. a Left column: To investigate spatial resolution, P1- decoding accuracy was calculated for subsampled electrodes (50 samples using Poisson disc sampling) at maintained coverage. Subsampled electrode populations with resolutions less than 1.5 mm crossed the 95% threshold. The decoding performance is improved by 17% and 29% with respect to 4-mm and 10-mm pitch, respectively. **Middle column:** To test for spatial coverage, decoding performance was assessed each rectangular subgrid at each possible array location. P1 accuracy increased with increasing coverage for both S1 and S2, and the maximum performance was achieved at the widest coverage possible. **Right column:** To examine the effect of micro-scale sampling, P1 accuracy was calculated on spatially averaged neural data with smoothing windows of varying grid-sizes. Decoding performance was maximal with the micro-scale contact size (200 µm) and decreased with increased spatial smoothing (i.e., increased synthetic contact size). The 95% max-accuracy thresholds and theoretical chance boundaries were drawn separately for each subject. **b** Median decoding scores indicate that the influence of spatial-resolution, coverage, and micro-scale contact is stable for phonemes across all positions for both S1 and S2. **c** Effect of spatial-resolution on the ability to decode the number of unique phoneme labels (N) for both S1 (violet) and S2 (green). Spatial resolution required to achieve 90-95% of maximum decoding performance increased with increase in N and saturated to resolutions lower than 1.5 mm for N greater than 5. Thus, the analysis demonstrated the necessity of spatial-resolution, spatial coverage, and micro-scale sampling for accurate speech decoding.

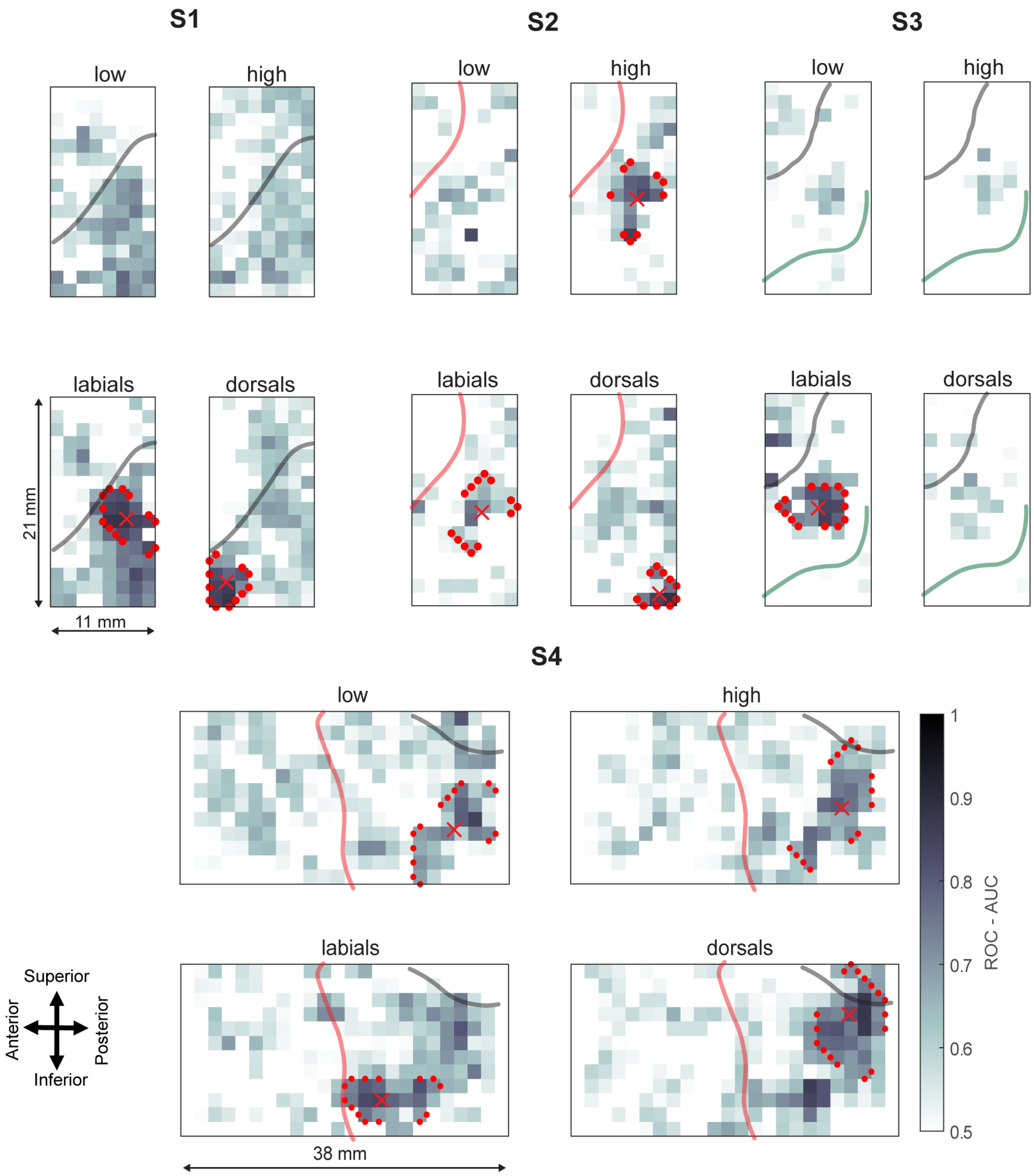

Supplementary Figure 15. **Articulator encoding maps for high-resolution neural recordings**. Univariate decoding of four articulatory features (low vowels, high vowels, labial consonants, dorsal consonants) identified articulatory tuning for each electrode. The spatial arrangement of resultant AUC values shows articulatory feature maps for all subjects. These maps showed distinct spatial clusters within the array, as identified by red contours, which identify the 90th percentile of AUC values across all articulators (colored marker x indicates the corresponding centroids). Shaded red, grey and green curves indicate the locations of pre-central, central, and post-central sulcus, respectively.

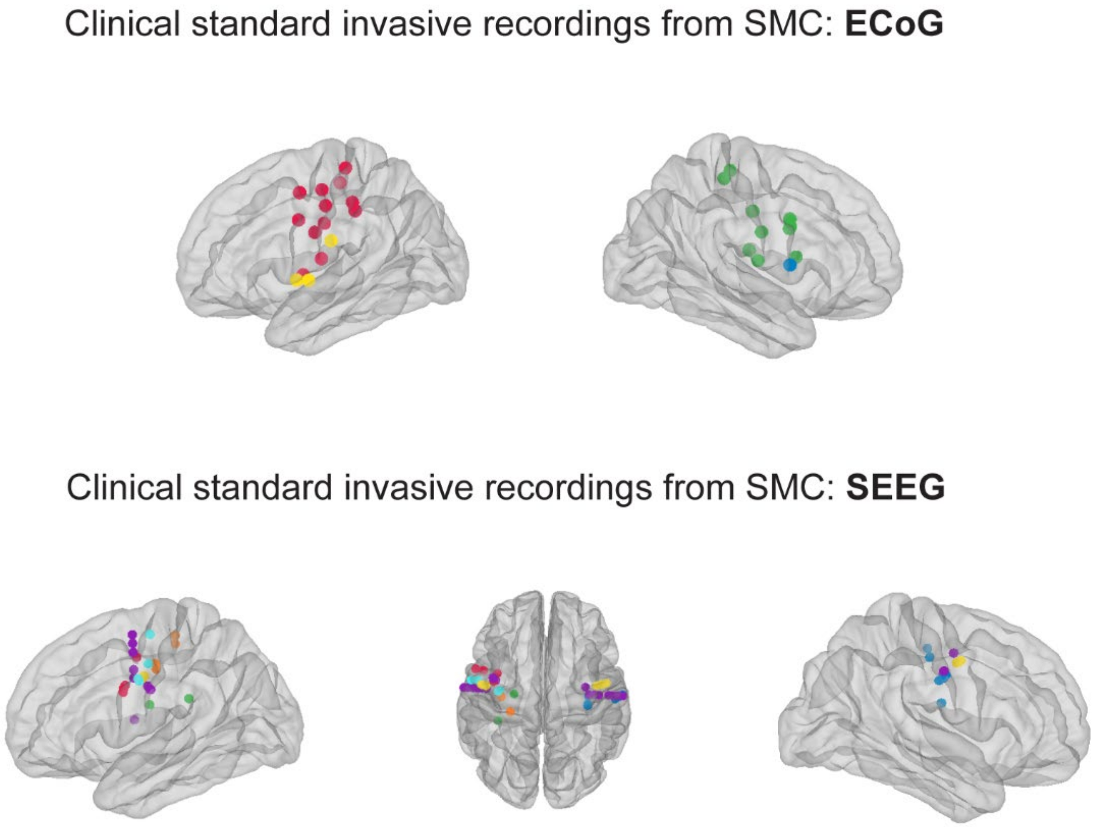

Supplementary Figure 16. Locations of pre-operative IEEG recordings in subjects with epilepsy during clinical monitoring. Each color marking indicates the location of an IEEG electrode (ECoG or SEEG) over sensorimotor cortex. The electrodes are color-coded with respect to subject (ECoG: 4, and SEEG: 7), and exhibited significant activation in the HG band during speech.

Supplementary Table 1:

Clinical summary of patients: Intra-operative awake surgery

Patient	Diagnosis	Electrode type/ No. of recording electrodes (coverage)	Electrode material	Number of electrodes with significant HG activation in SMC (estimated coverage)
S1	Parkinson's	μECoG: 128 (231 mm^2)	Gold	111 (199 mm^2)
S2	Parkinson's	μECoG: 128 (231 mm^2)	Platinum-Iridium	111 (199 mm^2)
S3	Parkinson's	μECoG: 128 (231 mm^2)	Platinum-Iridium	60 (108 mm^2)
S4	Tumor resection	μECoG: 256 (798 mm^2)	Platinum-Iridium	149 (440 mm^2)

Mean age: 53
Total number of females: 1

Clinical summary of patients: Pre-operative epileptic monitoring

Patient	Diagnosis	Electrode type: Total No. of recording electrodes	Electrode spacing s (mm)	Number of electrodes with significant HG activation in SMC (estimated coverage)
D6	Epilepsy	Ad-Tech 64 channel ECoG: 96	10	12 (*1200 mm^2)
D7	Epilepsy	Ad-Tech 64 channel ECoG: 102	10	10 (*1000 mm^2)
D24	Epilepsy	Ad-Tech 48 channel ECoG: 52	10	3 (*300 mm^2)
D26	Epilepsy	Ad-Tech 48 channel ECoG: 60	10	1 (*100 mm^2)
D35	Epilepsy	PMT SEEG: 174	3.5	4 (**38.4 mm^2)
D40	Epilepsy	PMT SEEG: 188	3.5	2 (**19.2 mm^2)
D52	Epilepsy	PMT SEEG: 194	3.5	5 (**48.1 mm^2)
D55	Epilepsy	PMT SEEG: 190	3.5	6 (**57.7 mm^2)
D59	Epilepsy	PMT SEEG: 184	3.5	4 (**38.5 mm^2)
D60	Epilepsy	PMT SEEG: 252	3.5	20 (**192.4 mm^2)
D70	Epilepsy	Ad-Tech SEEG: 202	5	4 (**78.5 mm^2)

* Estimated coverage for ECoG: $A = n * s^2$;

** Estimated coverage for SEEG: $A = n * \pi * \left(\frac{s}{2}\right)^2$; n – number of electrodes, s – electrode spacing

Mean age: 30
Total number of females: 7

Supplementary Table 2: Stimulus labels for speech repetition task

CVC	VCV
/bab/	/abae/
/baek/	/abi/
/bak/	/aeba/
/bup/	/aebi/
/gab/	/aebu/
/gaeb/	/aega/
/gaev/	/aeka/
/gak/	/aepi/
/gav/	/aka/
/gig/	/aku/
/gip/	/ava/
/gub/	/avae/
/kab/	/ibu/
/kaeg/	/ika/
/kub/	/ikae/
/kug/	/ipu/
/paek/	/iva/
/paep/	/ivu/
/paev/	/uba/
/puk/	/uga/
/pup/	/ugae/
/vaek/	/ukae/
/vaeg/	/upi/
/vip/	/upu/
/vug/	/uvae/
/vuk/	/uvi/

Supplementary Table 3: Neural data duration for each intraoperative patient

Patient	Blocks/Trials	Total spoken duration (minutes)	Number of correct trial repetitions (n)	Neural data duration (minutes; 1s per trial)<br>T = n * 1/60	Neural data duration to train SVD-LDA (minutes; 20-fold CV)<br>0.95 * T	Neural data duration to train seq2seq RNN (minutes; 80% training held out)<br>0.8 * T
S1	3/156	1.04	149	2.48	2.36	1.98
S2	3/156	1.15	152	2.53	2.4	2.02
S3	3/156	1.45	153	2.55	2.42	N.A.
S4	1/52	0.47	52	0.87	0.83	N.A.

N.A. – not applicable

Supplementary Table 4: Articulator coverage and tuning

Subjects		Low	High	Labials	Dorsal
S1 (16 x 8)	coverage (mm^2)	~	~	72.5	17.7
	mean AUC	~	~	0.9	0.9
	Centroid coordinates* (X, Y)	~	~	(9.8, 6.2)	(14.7, 1.7)
S2 (16 x 8)		~	26.5	28.3	10.6
		~	0.88	0.82	0.91
		~	(9.1, 5.4)	(9.2, 5.6)	(15.4, 7.2)
S3 (16 x 8)		~	~	47.8	~
		~	~	0.81	~
		~	~	(8.9, 4.6)	
S4 (12 x 22)		79.88	85.8	56.2	79.88
		0.85	0.8	0.84	0.88
		(8.5, 20.3)	(7.2, 20.3)	(9.8, 15.6)	(4.6, 21.1)

*top-left corner as origin
~ spatial clustering not observed

Supplementary Table 5: Hyperparameters for seq2seq based encoder-decoder model

**Hyperparameters**	**S1**	**S2**
Number of convolutional filters	100	90
RNN units	800	900
l2 penalty	1e-6	1e-5
1D convolutional filter length	10	
Number of training epochs	800	
Learning rate	1e-3	
Optimization	Adam	

Supplementary Table 6: Example decoded phoneme sequences using seq2seq based encoder-decoder model

S1

True	Predicted
'/vuk/'	'/vuk/'
'/aeka/'	'/aeka/'
'/gip/'	'/gip/'
'/gub/'	'/gub/'
'/puk/'	'/pup/'
'/baek/'	'/vaek/'
'/baek/'	'/aeaek/'
'/abae/'	'/bbae/'
'/vuk/'	'/puk/'
'/uba/'	'/aeba/'
'/uvae/'	'/ubae/'
'/bup/'	'/vup/'
'/vug/'	'/vuk/'
'/vaek/'	'/vak/'
'/bak/'	'/aak/'
'/ika/'	'/aeaa/'
'/uvi/'	'/uak/'
'/gaeb/'	'/kab/'
'/uga/'	'/aeba/'
'/gaev/'	'/kaeg/'
'/uba/'	'/upu/'
'/ava/'	'/aeba/'
'/paev/'	'/vaek/'
'/aka/'	'/aega/'
'/upi/'	'/vui/'
'/aku/'	'/aga/'
'/paep/'	'/bab/'
'/gaeb/'	'/ipi/'
'/upu/'	'/aba/'
'/paek/'	'/aka/'

S2

True	Predicted
'/gaev/'	'/gaev/'
'/gip/'	'/gip/'
'/gak/'	'/gak/'
'/ibu/'	'/ibu/'
'/abae/'	'/abae/'
'/kab/'	'/kab/'
'/kub/'	'/kub/'
'/bab/'	'/baa/'
'/vug/'	'/gug/'
'/baek/'	'/baep/'
'/gig/'	'/gib/'
'/upi/'	'/ipi/'
'/vip/'	'/pip/'
'/gip/'	'/pip/'
'/ava/'	'/aba/'
'/aka/'	'/aeka/'
'/paek/'	'/vaek/'
'/gab/'	'/bab/'
'/uvae/'	'/aevae/'
'/upu/'	'/uku/'
'/uga/'	'/aeka/'
'/avae/'	'/aebae/'
'/ipu/'	'/ubu/'
'/gav/'	'/gub/'
'/paev/'	'/aeaeae/'
'/puk/'	'/vug/'
'/aeka/'	'/aebae/'
'/aku/'	'/ubu/'
'/uvae/'	'/aba/'
'/ipu/'	'/ugae/'
'/iva/'	'/aeaeae/'
