# Supplementary material for: High-resolution neural recordings improve the accuracy of speech decoding
Source: Nat Commun. 2023 Nov 6;14:6938. doi: 10.1038/s41467-023-42555-1 (PMC10628285; doi:10.1038/s41467-023-42555-1)
Supplement: Supplementary file 3 — Reporting Summary [file 41467_2023_42555_MOESM3_ESM.pdf]

nature portfolio

Corresponding author(s): Gregory Cogan, Jonathan Viventi

Last updated by author(s): Oct 2, 2023

Reporting Summary

Nature Portfolio wishes to improve the reproducibility of the work that we publish. This form provides structure for consistency and transparency in reporting. For further information on Nature Portfolio policies, see our Editorial Policies and the Editorial Policy Checklist.

Statistics

For all statistical analyses, confirm that the following items are present in the figure legend, table legend, main text, or Methods section.

n/a	Confirmed	
☐	☒	The exact sample size (*n*) for each experimental group/condition, given as a discrete number and unit of measurement
☐	☒	A statement on whether measurements were taken from distinct samples or whether the same sample was measured repeatedly
☐	☒	The statistical test(s) used AND whether they are one- or two-sided<br>*Only common tests should be described solely by name; describe more complex techniques in the Methods section.*
☐	☒	A description of all covariates tested
☐	☒	A description of any assumptions or corrections, such as tests of normality and adjustment for multiple comparisons
☐	☒	A full description of the statistical parameters including central tendency (e.g. means) or other basic estimates (e.g. regression coefficient) AND variation (e.g. standard deviation) or associated estimates of uncertainty (e.g. confidence intervals)
☐	☒	For null hypothesis testing, the test statistic (e.g. *F*, *t*, *r*) with confidence intervals, effect sizes, degrees of freedom and *P* value noted<br>*Give P values as exact values whenever suitable.*
☒	☐	For Bayesian analysis, information on the choice of priors and Markov chain Monte Carlo settings
☒	☐	For hierarchical and complex designs, identification of the appropriate level for tests and full reporting of outcomes
☐	☒	Estimates of effect sizes (e.g. Cohen's *d*, Pearson's *r*), indicating how they were calculated

Our web collection on statistics for biologists contains articles on many of the points above.

Software and code

Policy information about availability of computer code

Data collection	We used Intan RHX Data Acquisition Software (Version 3.0) for intra-operative recording, and Natus NeuroWorks EEG software for pre-operative clinical recording. All of our speech tasks were designed using Psychtoolbox scripts in MATLAB 2014a.
Data analysis	All of our data analysis were performed using the following softwares: MATLAB 2021a, Python 3.7, Tensorflow 2.0, and Keras 2.4. We also used custom toolboxes for high-gamma extraction (https://github.com/Naplib/Naplib) and hyperparameter optimization (https://keras.io/keras_tuner/).<br>The MATLAB files to perform decoding are available through Zenodo at<br>Suseendrakumar Duraivel. coganlab/micro_ecog_phoneme_decoding: v1.0. (2023) doi:10.5281/zenodo.8384194.

For manuscripts utilizing custom algorithms or software that are central to the research but not yet described in published literature, software must be made available to editors and reviewers. We strongly encourage code deposition in a community repository (e.g. GitHub). See the Nature Portfolio guidelines for submitting code & software for further information.

Data

Policy information about availability of data

All manuscripts must include a data availability statement. This statement should provide the following information, where applicable:

- Accession codes, unique identifiers, or web links for publicly available datasets
- A description of any restrictions on data availability
- For clinical datasets or third party data, please ensure that the statement adheres to our policy

The data from µECoG recordings that support the decoding analysis are available via the Data Archive for The Brain Initiative (DABI; accession code: https://dabi.loni.usc.edu/dsi/7RKNEJSWZXFW) under restricted access for PHI and IRB restrictions. The access can be obtained from the corresponding authors (gregory.cogan@duke.edu, j.viventi@duke.edu). Source data are provided with this paper.

Research involving human participants, their data, or biological material

Policy information about studies with human participants or human data. See also policy information about sex, gender (identity/presentation), and sexual orientation and race, ethnicity and racism.

Reporting on sex and gender	Participants in the intra-operative study were 61, 63, 62, and 26 years old (male, male, male, and female respectively). Participants in the in-unit extraoperative setting had mean age of 30 (4 males and 7 females). Sex and gender based analysis were not performed in this study.
Reporting on race, ethnicity, or other socially relevant groupings	All participants were native speakers of English.
Population characteristics	Participants in the intra-operative settings (mean age: 53, 1 female patient) were all candidates for neurosurgery within the Duke University Health system. Three of the four participants were diagnosed with Parkinson's disease and required DBS implantation. The fourth participant was a surgical candidate for tumor resection.<br>Participants in the in-unit extraoperative setting (mean age: 30, 7 female patients) were diagnosed with intractable epilepsy and required pre-operative epileptic monitoring for surgical planning.
Recruitment	Participants were recruited to participate in the research study prior to surgery. Informed consent was obtained at this time. The participants were informed that their decision to participate in the research study was strictly voluntary, and would not impact their clinical treatment. All participants were native speakers of English, and had no speech impairment issues, therefore, we did not expect self-selection biases in our study.
Ethics oversight	All our research studies were approved by the Institutional Review Boards of the Duke University Health System under the following protocol IDs:<br>Pro00072892: Studying human cognition and neurological disorders using µECoG electrodes<br>Pro00065476: Network Dynamics of Human Cortical Processing

Note that full information on the approval of the study protocol must also be provided in the manuscript.

Field-specific reporting

Please select the one below that is the best fit for your research. If you are not sure, read the appropriate sections before making your selection.

☒ Life sciences ☐ Behavioural & social sciences ☐ Ecological, evolutionary & environmental sciences

For a reference copy of the document with all sections, see nature.com/documents/nr-reporting-summary-flat.pdf

Life sciences study design

All studies must disclose on these points even when the disclosure is negative.

Sample size	Speech and neural recordings collected for each participant was based on the participant pool from surgical patients and the amount of time was based on surgical considerations as well as the amount of time each participant was willing to volunteer for the study. The data required were sufficient to perform speech decoding.
Data exclusions	Speech trials with substantial noisy neural recordings and trials excluded from analysis (see Methods). We also removed trials where patients incorrectly repeated the stimulus.
Replication	We performed analysis independently on each participant to confirm patient-specific findings. Within subjects, the offline decoding analysis involved nested cross-validation, where decoding models were trained on different instances of data, that mimics replicability. Across patients, the highest decoding performance was consistent between two patients (S1 and S2) that demonstrates reproducibility of results.
Randomization	Not applicable for the study due to the clinical basis of the participant pool.

Blinding	Not applicable for the study due to the clinical basis of the participant pool.

Reporting for specific materials, systems and methods

We require information from authors about some types of materials, experimental systems and methods used in many studies. Here, indicate whether each material, system or method listed is relevant to your study. If you are not sure if a list item applies to your research, read the appropriate section before selecting a response.

Materials & experimental systems

n/a	Involved in the study
☒	☐ Antibodies
☒	☐ Eukaryotic cell lines
☒	☐ Palaeontology and archaeology
☒	☐ Animals and other organisms
☒	☐ Clinical data
☒	☐ Dual use research of concern
☒	☐ Plants

Methods

n/a	Involved in the study
☒	☐ ChIP-seq
☒	☐ Flow cytometry
☒	☐ MRI-based neuroimaging
